# Exploring men’s use of mental health support offered by an Australian Employee Assistance Program (EAP): perspectives from a focus-group study with males working in blue- and white-collar industries

**DOI:** 10.1186/s13033-021-00489-5

**Published:** 2021-08-04

**Authors:** Lynda R. Matthews, Jacky Gerald, Glenda M. Jessup

**Affiliations:** 1 School of Health Sciences, Faculty of Medicine and Health, The University of Sydney, Camperdown, NSW 2006 Australia; 2 Acacia Connection EAP, Level 1, 31 Merivale Street, South Brisbane, Qld 4101 Australia

**Keywords:** Employee Assistance Programs, Men’s health, Mental health, Mental illness, Human Resource Management, Masculinity, Gender, Workplace services, Men

## Abstract

**Background:**

Men continue to be overrepresented in the Australian suicide statistics despite wide scale public health initiatives to improve men’s mental health literacy and to increase their help-seeking behaviour. Employee Assistance Programs (EAP) deliver free and confidential mental health support; however, their services are underutilised by men. In the absence of contemporary literature that explores end-user experiences of EAPs, we asked men from blue- and white-collar employment settings about the barriers and enablers to using EAP services and explored differences between employment settings.

**Methods:**

Forty-four men participated in this qualitative study: 32 from one white-collar employer and 12 from one blue-collar employer. Two qualified mental health professionals facilitated five first-round and three second-round focus groups and one interview with white-collar workers, and two focus groups and three interviews with blue-collar workers. Data were thematically analysed using a framework approach.

**Results:**

Four of the six main themes were barriers: no need for EAP—alternative supports; uncertainty of EAP services; scepticism and distrust of EAP; and societal and workplace cultures. Elements of enduring barriers to EAP use were contained within sub-themes. These included lack of knowledge about EAPs, issues of trustworthiness and confidentiality, and fear of stigma and career jeopardy. Enablers comprised the need for attractive, reliable messaging and proactive connections and service delivery. Differences within sub-themes for white-collar and blue-collar groups reflected the corporate nature of work and workplace culture for white-collar participants, and workers’ communication and practical problem resolution preferences for blue-collar workers.

**Conclusion:**

Some elements identified in the barriers to EAP use are more entrenched than were previously estimated and these need to be a priority for action to increase confidence in EAP services by end-users. EAPs that have a visible and proactive presence in the workplace, that tailor their marketing and service delivery to different workgroups, that provide a competitive advantage to its service users, and more confidently conveys independence from its client organisations may help to increase men’s interest in accessing EAP support services. Further initiatives that reduce the stigma surrounding mental health and help-seeking both in society and the workplace are needed.

## Introduction

In Australia, approximately 76% of all deaths by suicide in Australia are men [1]. It is a striking statistic and one that is not limited to Australia. Suicide accounts for approximately 1.4% of all deaths worldwide [2] and in most regions men are over-represented in the statistics, particularly in high income countries such as those in the Americas, Europe and Western Pacific regions [3]. Early identification, treatment and care of people experiencing acute emotional distress, mental health and substance use disorders is one of the key components of suicide prevention [4]. However, many men who need care for distress or mental health issues do not seek help [5–9]. There has been a strong focus in Australia on developing wide scale public health initiatives to improve men’s mental health literacy, to increase help-seeking behaviour, and to reduce the rates of suicide (for examples, see [10, 11]). On the whole, few have been successful in doing so [5, 12]; the rates of suicide have not declined over the past decade [1] and for every suicide death, as at least 20 individuals will attempt suicide [2, 7].

There is increasing interest in the use of a workplace settings approach to men’s suicide prevention because most deaths by suicide are among people of working age [1]. Examples of successful mental health and suicide prevention programs based in workplace settings include Mates in Construction [13], Farm Link [14], IncoLink [15], and OzHelp Foundation [16]. These programs are organised around specific at-risk occupational groups, apprentices, and blue-collar workers in male dominated professions with workplaces that have historically reinforced traditional masculine norms of strength, stoicism, and emotional restriction. The results of those programs evaluated suggest that these targeted prevention initiatives have beneficial effects [17]. They have been particularly successful because of their use of industry-wide education and training, a buddy system, and/or training gatekeepers who identify workers at risk and refer for treatment [17].

Employee Assistance Programs (EAPs), an employer-funded service for employees and their families, also deliver mental health and suicide prevention training in the workplace. EAPs provide a range counselling and support services that are designed to assist individuals in the work context, promote individual health, and diminish the chances of personal or work problems negatively impacting the work environment [18]. They are considered an important part of an organisation’s occupational health and safety and wellness initiatives [18]. EAPs are available to employees in approximately 80% of Australia’s top 500 companies and many other small to medium organisations [19] and over 75% of US public sector and 40% of private sector organisations [20]. The widespread use of EAPs by companies means that they have an extensive reach to working age men in a range of organisations and industries, and therefore provide an existing professional mental health service delivery framework from which to assist men.

In a recent systematic review that identified the effectiveness of a range of EAP interventions [21], five of the 17 studies reviewed examined effectiveness on personal functioning [22–26]. These studies reported significant improvement in depressive symptoms [26], clinical distress [25], and psychosocial function [22–24]. None of the studies were conducted in Australia. Although few EAPs have examined the impact of their suicide prevention programs for users, a systematic review of workplace suicide prevention activities [17] identified one Japanese EAP study where a significant decrease was reported in the levels of self-reported depression and suicidal thoughts in men between baseline measures and the 2-year follow-up period [26].

EAP services have demonstrated evidence of success, yet like community-based mental health services they are under-utilised by males, a trend that has been reported over time (see, for example [18, 27–30]). Studies examining the use of EAPs by combined samples of male and female employees have suggested that low uptake by employees is due to their lack of knowledge about the existence of EAPs and also about the range of services provided by EAPs [30–32]. Concerns over confidentiality of the service, particularly in relation to supervisors or managers becoming aware of staff using the service, has also been identified as impeding EAP use [33]. However, it is uncertain whether the barriers reported by combined samples are the only obstacles to men’s use of workplace EAP services. It is also unclear as to whether findings from research conducted in different countries can be applied to EAPs in Australia. Previous research [34] suggests that Australia’s EAP service delivery model is a “some-what unique service-delivery model, grounded in local legislative and industrial influences” (p. 353).

The wider literature reporting barriers to men’s use of non-workplace based mental health services identifies several factors that may also apply to use of EAP services. These include the stigma attached to mental health service use [35], men’s beliefs about mental health [5, 9, 36], men’s perceptions of the low relevance of services to their needs [36], traditional masculine norms that influence attitudes about seeking treatment [5, 37–39], services not being culturally appropriate, and socio-economic barriers to seeking care [10, 38].

The socio-economic barriers to seeking care is of particular interest to men’s use of EAP services because workers in the lower skilled occupations (blue-collar workers), who are most likely to have limited social and economic resources and access to health care, have also been found to be at greater risk of suicide than the highest skill-level group (white-collar workers) [40]. Studies have pointed to the role of psychosocial stressors in the workplace, such as low control over work, monotony of work, and high psychological demands, and their relationship with suicide [41, 42]. In this context, workplace based EAP services are well placed to address the consequences for workers of psychosocial stressors. Being provided at no cost to users, EAPs have no apparent socio-economic barriers to their use and would seem to play an important role in helping to reduce workers’ distress [21].

Considering this ‘stepwise gradient in risk’ for suicide within occupational groups [40, p409] we were interested in finding out whether men in blue- and white-collar work in Australia shared the same perspectives on EAP use. We could find little contemporary literature that helped us answer this question. Further, much of the literature exploring the use of EAP services is dated. Economic and social development in the past decade has resulted in new work arrangements, increased hours and pace of work, and new psychosocial work hazards [43]. These changes have resulted in a broadening of EAP services to meet the needs of workers and their families as well as organisational needs [44]. Therefore, the relevance of findings from older studies to today’s workers is unknown.

Accordingly, the aims of this study were to explore the obstacles and enablers to men’s use of an Australian EAP mental health support service and to determine whether perspectives differed based on employment in blue- or white-collar organisations. Specifically, this exploratory research was guided by the following research questions:

1. Why don’t men seek help from their EAP service?
2. What would encourage men to do so?
3. Does the situation differ for men working in blue-collar versus those working in white-collar organisations?

## Methods

### Participant recruitment

Client organisations of Acacia Connection EAP, a national provider of EAP services in Australia and member of the Employee Assistance Professional Association of Australasia, were advised of the research through a presentation at a regular client meeting. Subsequent to the meeting, two organisations—one metropolitan-based manufacturing firm (blue-collar), and one central city and metropolitan-based accounting firm (white-collar)—expressed interest in participating in the research. Both organisations provided written agreement to the EAP provider and the University for their male employees to be sent an invitation to participate in focus groups or interviews to discuss men’s use of EAPs. Following approval of the study’s protocol by the University of Sydney’s Human Research Ethics Committee (#2016/820), the organisations disseminated written information about the study to all adult male employees. The written information included an invitation to participate in a focus group, or interview if they were unable to attend a focus group, and a Participant Information Sheet that provided plain English details about the study. The letter asked that interested male employees contact the research team directly for additional information, or to volunteer to participate. No financial incentives were provided for participation. Fifty-two men provided written consent to participate in the study and 44 attended the focus groups or interviews. Five who did not participate cited intervening work events, two did not present for the groups, and one had left employment with the organisation.

### Procedure

This study is presented in line with the consolidated criteria for reporting qualitative studies (COREQ) guidelines [45]. All participants provided written consent prior to attending the focus groups or interviews. They were also asked to self-report descriptive information including age, relationship status, number of dependents, postcode, and role in the organisation.

Two experienced and qualified mental health professionals facilitated the focus groups and interviews over a period of three months (December 2017–February 2018). Each focus group was allocated one hour to align with a lunch break, however at the end of the hour many of the men in the white-collar groups still had contributions to make so a second round of focus groups was offered for those who wanted to attend. Many of the issues identified in the first round were revisited with more detail in the second round suggesting saturation of data. However, as we included all individuals who had volunteered to participate in the focus groups, data saturation was not a consideration at the time.

All groups were held onsite at each organisation in a room that was separate to the main workplace; only participants and facilitators were present. With participants’ written consent the focus groups and interviews were audio recorded. Interviews were conducted in person for the white-collar participants and in person and by phone for blue-collar participants. One facilitator was an experienced PhD-qualified researcher and mental health professional not known to the organisations or participants. The other facilitator was a qualified and experienced counsellor and a state manager of the EAP provider for the client organisations. She was known to some, but not all, of the participants. In the introduction to the groups and interviews, her honesty in discussing the genesis of the study—the need to find ways to reduce the high suicide rates in working aged men—developed rapid rapport and openness in the men’s discussions.

A semi-structured focus group guide was used as a basis for discussion. Participants were asked about their knowledge of EAPs, their use of EAPs, and about enablers and barriers to their use of EAPs. Prompts were used to facilitate in-depth discussion to avoid prescriptive questioning and brief notes were taken by one of the facilitators during discussions. The recorded data were transcribed verbatim and de-identified. Transcripts were not returned to participants because they did not individually identify each participant.

### Analysis

**Fig. 1:**
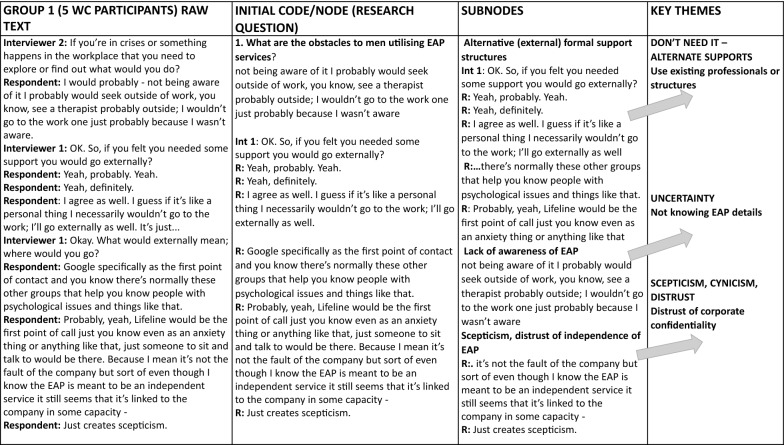
Thumbnail overview of analysis process

Data were analysed using a framework approach [46], which is suited to research with specific research questions and a primary focus on describing and interpreting experiences in specific situations [47]. The transcripts were read and initially analysed by two members of the research team for purpose of familiarization and interpretation. The third member of the team then listened to the audio files and read the transcripts. The transcribed data were then entered into NVivo 12 [48] and each interview was analysed separately. The first interview was coded using the first two research questions (why don’t men use EAPs? what would encourage them to do so?) as the initial NVivo nodes (codes) (see Fig. 1 example). Data from the second and subsequent interviews were coded into the existing sub-nodes, and new sub-nodes were created as needed. When a new sub-node was introduced, all previous transcripts were reviewed again to ensure that all relevant data were included. This process generated an index of broad descriptive categories.

To improve trustworthiness and consistency of the analysis, a more detailed analysis that focused on a synthesis of recurring patterns within and across categories was then undertaken to create the key themes that characterised common dimensions of men’s perspectives of EAPs [see 49]. Each member brought different professional perspectives to the interpretation: psychotherapy, mental health rehabilitation, and social psychology. Differences in data interpretation were discussed and resolved collaboratively by the team, thus enhancing dependability and conformability [50].

Quotes that were most representative of the themes were included in the results as exemplars of evidence. The number in parentheses after each quote represents the focus group or interview number.

## Results

### Participants

Forty-four male employees participated in the study; 12 men were blue-collar workers, and 32 men were white-collar workers. Table 1 provides the demographic information for participants. The average age of the total group of participants was 37.37 years (*SD* = 10.94; range 22–60) and all levels of management were represented in the data collected. There were no significant differences between sectors in the number of dependents (Fishers exact test = 0.249), relationship status (Fishers exact test = 0.279), or percentage of each management role participating in the groups (Fishers exact test = 0.416), although participants from the white-collar sector were significantly younger than those from the blue-collar sector (*t* = − 3.281, *df* = 38, *p* = 0.002). The men participated in a total of 10 focus groups and 4 interviews (see Table 2), ranging in length from 11 to 65 min. White-collar workers participated in six first-round, two follow-up (round 2) focus groups and one individual interview. Blue-collar workers participated in two first-round focus groups and three individual interviews. One participant disclosed he had attempted suicide some years previously and four others said they had male friends who had died by suicide, some recently.

**Table 1:** Participant demographic information from focus groups

	All participants (N = 44)	White-collar participants (N = 32)	Blue-collar participants (N = 12)
Age, years M (SD)	37.37 (10.94)	34.48 (8.69)	46.67 (12.73)
Relationship, n (% N)			
Married	20 (45.45)	13 (40.62)	7 (58.33)
Partner	5 (11.36)	4 (12.50)	1 (8.33)
Single	12 (27.27)	11 (34.38)	1 (8.33)
Not provided	7 (15.9)	4 (12.50)	3 (25.0)
Has dependents, n (% N)	17 (38.64)	11 (34.38)	6 (50.0)
Role in organization, n (% N)			
Senior management	13 (25.55)	11 (34.38)	2 (22.2)
Middle management	8 (18.18)	7 (21.89)	1 (8.33)
No management	17 (38.64)	11 (34.38)	6 (50.0)
Not provided	6 (13.64)	3 (9.38)	3 (25.0)
Used EAPs previously, n (% N)	8 (18.18)	7 (21.89)	1 (8.33)

6 participants did not provide demographic data

**Table 2:** Details of focus groups and interviews

Group/interview	Type	Category	N^a^	Round	Length (mins)
1	Focus group	White-collar	5	1	62
2	Focus group	White-collar	4	1	51
3	Focus group	White-collar	9	1	65
4	Focus group	White-collar	5	1	61
5	Focus group	White-collar	4	1	38
6	Interview	White-collar	1	1	41
7	Focus group	White-collar	4	2	50
8	Focus group	White-collar	2	2	54
9	Focus group	White-collar	3	2	56
10	Focus group	Blue-collar	4	1	53
11	Focus group	Blue-collar	5	1	47
12	Interview	Blue-collar	1	1	11
13	Interview	Blue-collar	1	1	19
14	Interview	Blue-collar	1	1	16

^a^Total n does not equal total number of participants; some participants attended a repeat focus group

### Why don’t men seek help from their EAP service?

**Fig. 2:**
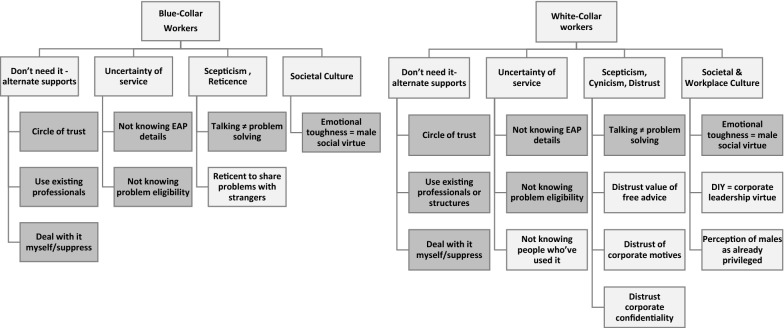
Barriers to EAP service access

Some participants did not know what an EAP was, and two advised that they had just recently been made aware that their current employer offered an EAP. Figure 2 provides an overview of the barriers to accessing an EAP for white- and blue- collar participants and illustrates points of similarity and difference. Barrier similarity is illustrated by the darker shading and these themes tended to be those more commonly discussed by participants.

#### Don’t need it: alternative pathways of support

Participants distinguished between levels of support needed for problems. Most participants stated that their first point of call for most problems was their informal “circle of trust’’, as some referred to it. In cases where sharing with their circle of trust had the potential to become problematic, depending on the nature of the problem, they would use as an alternative existing professionals or workplace support structures. Alternatively, they would deal with issues themselves, often by suppressing them.

*Circle of trust* This was the first point of call for most problems and could encompass both non-work and work friends, spouse or partner, or family. The blue-collar workers mentioned having drinking sessions with co-workers or mates as a way of coping with problems and supporting each other. While some participants felt able to share everything with their spouse, the nature of problems shared within a circle of trust could have limits. The men did not want to share information with friends or family if the problem concerned those friends or family, or if they felt embarrassed by actions they had taken: “Maybe say you've only got, like, one friend and maybe the issue is with them” (12). They also did not want to share information with partners or spouses that would imply they were not happy in their situation or that they felt could be damaging: “Can you imagine walking into the family home and saying: ‘Oh no, I can't do this any longer.’ You might as well put a match to the place” (8).

The limitations of the circle of trust were poignantly illustrated by one participant who had experienced suicidal behaviours years ago, feeling unable to share his burdens at the time: “I didn’t really have anybody to turn to and the only one I could have turned to would have been my mother, but I didn’t want to upset her … so she never knew” (14).

Within the white-collar workplace, the blurring of work and personal boundaries could create barriers to sharing personal information. Additionally, the transient nature and competitive mentality of the workforce meant some men never felt able to completely rely on the continued support of work colleagues.

It's never a ‘this is our relationship, we are friends and that's fixed and that's locked and loaded’… and by the corporate nature of it, it attracts people who are a little dog-eat-dog in some things, and don't suit well towards ‘we can be mates first then we can be colleagues second.’ (9)

*Existing professionals and structures* This alternative form of support came from existing professionals and structures such as the family doctor, personally known psychologists, or workplace wellbeing support systems. The only existing professional support mentioned by the blue-collar participants was that of a family doctor whereas white-collar participants had access to additional internal workplace wellbeing structures, including a system of employee coaching/mentoring. The white-collar participants said Human Resources did not promote EAP as a first point of call but directed employees to internal wellbeing structures.

They’ll go and point you to directions like ‘Have you thought about this [non-EAP attributed information] or tried it’ and you know it’s available on the intranet. (1)

The internal formal white-collar coaching and mentoring system however could be problematic in that coach and mentor roles were allocated on seniority rather than skill: “Well, everyone’s sort of like a coach or a counselling manager and partner, and for the most part… it’s a pretty useless relationship” (3). Not all senior managers welcomed, or knew how to manage, the information given to them in their mentoring role. Senior managers were, in turn, concerned as to what would happen to their own personal information.

… you can’t be certain on how they’re going to manage that information… so, it’s the walls up and say, ‘I won’t share it with these people’ because you don’t know how they’re going to manage the information (1).

*Deal with issues personally or suppress them* Rather than sharing problems, some participants said they preferred to deal with issues themselves or suppress them and hope that the issue would resolve in time.

Men find it embarrassing to ask for help, to confess that they do feel down. They feel a bit weak. It’s an uncomfortable topic to talk about and even when you do talk about it there’s a bit of awkwardness associated with it. So, we sort of try to tough it out and hope that it goes away. (6)

The men also acknowledged that by dealing with issues themselves they were taking a chance of allowing issues to get worse.

I'd rather just internalise it and deal with it rightly or wrongly how I see to deal with it… You pack it away, you deal with it, you deal with it, and then it gets bad. (10)

Men’s strategies for dealing with the problem themselves included alcohol consumption, humour or fitness: “They either get black-out drunk… Or the ones who are constructive about it like maybe do some sports or gym or something like that and try and—just like diversion…” (3). White-collar participants commonly used fitness and physical activity to reduce stress. Fitness was prominently promoted within their corporate wellness programs (including discount gym memberships and CEO endorsement) and was perceived as an easier, immediate, and more accessible option to deal with problems than was a largely unknown EAP service.

When you go through times of stress people always say just go to the gym, work it off, right, go to the gym, there’s no barrier to entry there mentally. You just go whenever you can. (5)

#### Uncertainty about the service

Participants’ not knowing what an EAP was, or not knowing its details, purpose and scope was a barrier to their utilising it. Problem eligibility and not knowing people who had used the service also contributed to this uncertainty.

*Not knowing EAP details* Many men did not know what the letters EAP stood for or what an Employee Assistance programme was. For those who did know, EAP was generally perceived as a service that was provided as part of their employment benefits but was intended to be independent of the workplace. Participants gave various responses as to its purpose or scope; many prefaced with, “I guess” or “I think”. These responses related to providing support: a solution, a sounding board and a widely held perception of EAP as a last resort for major issues when informal support systems were not, or no longer, viable.

Most participants were unaware that the service extended to family members or that they could request a counsellor from a specific cultural background. They were unaware of the different modes of access, or that it was available 24/7. Participants were also unaware of what would happen if they accessed the service. Who would they talk to? What would the process be?

It’s also people’s expectations of what they’re going to get…. Are they going in there to get an objective opinion from somebody? Are they going in there hopefully looking for advice from someone?... So, it’s I think just people understanding what they can get out of it. (7)

For white-collar workers, part of the reason for their lack of knowledge of an EAP was its information positioning. EAP information was either buried within induction materials or buried within an overwhelming amount of other “stuff.”

Starting a new job; you’re trying to get up-to-speed on learning how the job actually works… there’s heaps of other things like bank offers and deals with your mortgage and stuff that doesn’t apply to me, so I didn’t spend any time trying to look it up. (2)

EAP information was not differentiated enough from other white-collar corporate wellness programs or strategies. White-collar workers found it difficult to identify the personal benefit of the EAP, and to differentiate the EAP’s role and function from other facets of the existing corporate wellness structures.

That’s the way I've been perceiving the programme, that it’s something that I could suggest to people, rather than ah, I could benefit from it… it's very different in essence from the rest of the components of wellness. (5)

*Not Knowing Problem Eligibility* Uncertainly as to the magnitude of a problem worthy of an EAP, was a barrier to its use. This subtheme was a frequent response. Men were uncertain as to whether an EAP was for everyday problems or for something more serious.

You just said that “One in five of you in this room” which means one of us in this room... “would be undergoing a Mental Health issue right now, statistically,” right? I don’t even know what that is. We just have shit to deal with every day and I don’t think about it and go “I have a problem” or “I don’t have a problem” … I just have shit to deal with every day. (1)

In contrast to the perceptions that the EAP was only for issues or problems, one participant who had previously accessed an EAP said he had no defining issue to deal with: “I didn’t hit rock bottom or anything… it’s just like ‘I could feel better than I feel now’.” (4).

*Not knowing people who had used or recommended an EAP* This additional barrier was articulated by white-collar participants**.** These men said it could be hard to trust a service that was not recommended by someone they knew: “I don’t know anyone who’s used it. That sort of bothers me a little bit. Is it because it’s secret or is it because it’s not working?” (2).

They acknowledged that not knowing people who had used an EAP service may be due to the confidential nature of the service.

I can’t think of a leader that I’ve worked with that would have and has actively told somebody to call the EAP… that’s part of that confidentiality nature of people dealing with issues in a confidential approach. But you don’t see it overtly projected from our leaders in terms of the use and value of the system. (4)

#### Scepticism, cynicism, distrust

This barrier had five subthemes: (a) scepticism of the efficacy of ‘talking therapies’ to solve problems, (b) distrust of the value of free advice, (c) cynicism and distrust of corporate motives, (d) distrust of corporate confidentiality, and lastly (e) reticence to share personal problems with strangers (see Fig. 2).

*Scepticism of the efficacy of ‘talking therapies’* Both white-collar and blue-collar participants expressed their scepticism of EAP’s talking therapies: “I know it’s good to have someone to talk to but it’s just, you know, EAP’s not really at the forefront of the ways you deal with stress or burdens” (6)**.** Although men talked to their circle of trust, they perceived that what they would get from EAP professionals was counselling and viewed this as passively ‘talking’ about issues. They were not convinced that this approach—referred to as “warm fuzzies’’ (10) by some—could effectively address problems: “I think that's a male thing—you want a solution. You don't want to just go and talk about it” (10).

Participants’ habitual approaches to problems were to actively try and fix them, leave problems to resolve themselves, or bury the issues and not deal with them. Mere talking was not perceived as providing a solution: “Well what’s the point of talking to these people about this thing… how can they actually help me? I’m kind of sceptical of the actual value that would come out of it in an individual basis.” (4).

*Distrust the value of free advice* Some white-collar participants expressed a distrust of the value of the advice from an EAP. They perceived that because the service was free, it provided generic, rather than tailored, advice. They could not be confident of getting a good service.

If you’ve got something really potentially bad, wrong, then you don’t want to get the cheap-as-chips service that’s just available on a shelf. You want to make sure you get treated properly (7).

By way of contrast, a few white-collar participants who had used an EAP found the service different to their expectations. Their initial uncertainty about the quality of the service was dispelled as they realised that the EAP responded to their needs very well.

It wasn’t really that they gave me the answers… it’s more about listening and just asking you questions and helping you arrive at your conclusions. (3)

*Cynicism and distrust of corporate motives* White-collar workers also expressed cynicism and distrust of corporate motives for the provision of an EAP. They felt that the priorities of a corporation were the health of the business and its bottom line. The company unreasonably pressured employees to meet business targets while at the same time offering an EAP service.

And “here’s your targets and these are our expectations,” so, push the risk down to the individual. And then they worry why their wellbeing might not be good because they don’t know whether or not they’re going to advance in their career… what a bloke values, you know, which is security, which is social mobility, which is advancement and all those sorts of things, and you’ve got this… a firm who pushes all that risk down to you and then says, “Oh, we’ve got EAP program.” How am I supposed to believe that shit? (3)

Wellness was perceived as being addressed only in “lip-service” (5) or as a tick box to mitigate risks related to mental health. Some previous wellness strategies had not been perceived as employee friendly, so white-collar participants were not sure why the EAP services should be perceived differently.

*Distrust of corporate confidentiality* The strong perception of a business connection between the EAP provider and the employer underpinned an underlying distrust by white-collar participants of corporate confidentiality when using this employer-funded service. Many thought that others within the organisation would eventually know they were having an issue.

So, if you go “Well, I’ve got a work-related issue” this is work-related - you know this is an Employee Assistance Program; it all seems a little bit tied in too close together.… you can’t really disabuse yourself from that. (1)

*Reticence to share problems with strangers* Within the blue-collar groups, a limitation to sharing personal information outside the circle of trust was their reticence to share problems with strangers, people who knew nothing about them.

It's things you notice and maybe you just ask them [friends] a question or two and if they feel like talking to you, they will; and if they don't, they tell you to piss off—stronger words—but coming down to the fact that they know each other; you're never going to get that with a stranger because they wouldn’t know anything. (11)

#### Societal and workplace culture

The three subthemes underpinning this barrier were (a) social virtue in presenting as emotionally tough, (b) corporate virtue in dealing with issues yourself, and (c) perception of males as already privileged (see Fig. 2).

*Social virtue in presenting as emotionally tough* Both white-collar and blue-collar participants expressed this as a strong belief. It was variously expressed as a “male” thing, inculcated through upbringing. Solving difficulties themselves helped men toughen up, asking for help did not.

You have to try and overcome boundaries that men have been brought up to be, brought up to be hard all their life, right? That’s one of the hardest boundaries for them to break…They break it, they look [like] they’re being soft. [Reaching out] - it's not seen as a manly thing to do. (11)

Trying to resolve health problems without reaching out for help made you stronger and more resilient and was heeding the wisdom of forbears about what it is to be male.

You’re stressing-out and you don’t know where to deal with the issue, and in the back of my mind even as frustrated and tense as I was through the whole experience you go, “Oh, this is building resilience; this is good for me. You know this is going to make me better.” (1)

A corollary to this social virtue, was that an inability to resolve an issue themselves could cause men embarrassment or shame. Asking for help was a sign of softness or weakness and so men held onto their privacy and chanced the outcome.

Who wants to be the bloke that puts up his hand and goes, “oh, I can't cope.” There might be plenty of people that are sitting there thinking, “shit I wish I could.” But you don't want to be seen like that. (10)

Some participants had the belief that the younger generations of males would be more willing to seek help. Younger generations were thought to have improved mental health literacy from exposure to mental health public health campaigns and reaching out for help was something the fathers in the groups said they were attempting to teach their sons.

I think from when my grandparents were alive and with my parents - forget it, you've got to be tough, man up, suck it up and off you go. But I think as time goes past and what I pass on to my kids and what my kids pass on to their kids, I think the teachings will be different. (8)

*Dealing with issues yourself and problem-solving is a corporate virtue* In the corporate milieu, white-collar men needed to present “your best-est and strongest-self’’ (7). Thus, dealing with issues yourself and problem-solving was perceived to be a corporate virtue, particularly for men in, or aspiring to, leadership positions. It signalled to the individual and to peers and managers that they were capable. Corporate culture is competitive. Seeking help was not perceived as a corporate virtue, but as weakness. Any sign of weakness, particularly in the mental health area could be a stigma, rather than a sign of a leader.

You need to demonstrate that you can handle things. Having to pick up the phone or utilise one of these services is sometimes, there’s that mental block, that that’s a sign of weakness. Then as soon as that sign of weakness is exposed, people are going to be dubious about my capabilities… there’s that little chink in the armour and maybe you’re not as capable, not as good, not as fit to deliver as what should be. (4)

Knowledge that men had needed help for working through mental health issues was often kept private, although colleagues tended to find out about it by gossip or speculation. It was perceived as potentially stigmatising, as jeopardising long-term career advancement, perhaps not openly but by stealth.

If you work in a quite stressful team and it gets very tough at times... if you start showing you're weak, maybe people do think that has this guy got the potential to go to the next level when he’s struggling with this? (5).

*Perception of males as already “Privileged”* Some white-collar participants commented on the impact on them of a social discourse which promoted the empowering of women and characterised males as already privileged: “You hear about all these initiatives around the disparity of women. Women in the workplace. There’s no—it’s almost bad to say like ‘Men’ in the workplace” (7).

Participants were aware of corporate strategies to advance and empower women or people from minority groups and had thus deduced that their well-being was not seen as important. They need not be utilising resources (such as EAP) that people from minority groups could benefit from. Instead, they “suffer in silence’’ (8).

### What would encourage men to use an EAP?

**Fig. 3:**
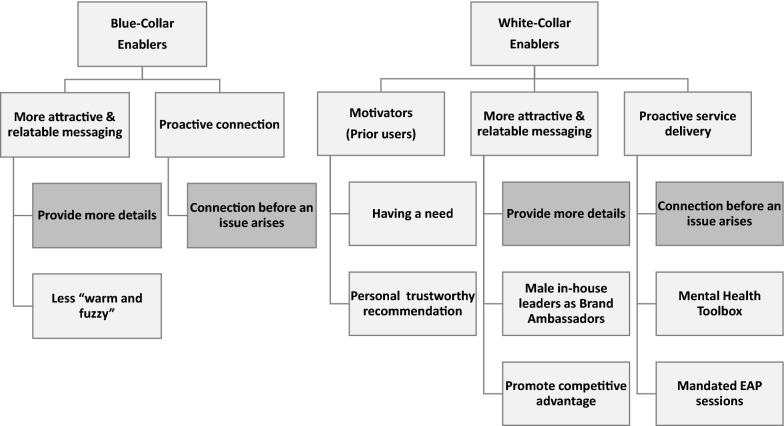
Enablers and motivators of EAP access

This section reports on motivators of actual EAP use (where disclosed) as well as what participants said would potentially motivate or enable them to use a service. Figure 3 provides an overview of the enablers and motivators of access to an EAP for white-collar and blue-collar participants. It also illustrates the points of similarity and difference (darker shading).

### Motivators and enablers of actual EAP use

A small group of participants disclosed that they had previously used EAP services with either their current or former employer. Their perspectives are described in line with COREQ guidelines [45] to report diverse cases and minor themes. One participant, the sole blue-collar worker to access an EAP, provided no detail, other than it was with a previous employer. The results for this section are therefore based on information from the white-collar participants. Table 3 lists the reasons for their previous use. These motivators and enablers (where disclosed) could be categorized as: (a) having an issue or need, and (b) having a personal recommendation by someone trustworthy (see Fig. 3).

**Table 3:** Motivators to use EAPs by prior users

Group	Motive/reason	How accessed
1	“help with some ways to think about stuff’”	Recommended by friend
2	Accidental death of a team member	Sessions as part of team referral (previous employer)
2	“I’ve had this event in my life. I wonder if [Company] offers support.”	Independently searched
3	Anxiety issues	Recommendation of counselling manager (senior colleague)
4	“no defining moment … I think I could feel better than I feel now”	Independently searched
5	Needed to speak to someone	Intranet search and colleague recommendation
7	“needed to talk to somebody ASAP”	Independently searched
10	Not disclosed	Not disclosed (previous employer)

#### Having an issue or a need

For some participants, this need was precipitated by an event. Another participant had an issue he had disclosed to a senior colleague. Others needed to talk or needed help with their thinking, or (as noted earlier) to feel better.

#### Personal Recommendations of Someone Trustworthy

While some participants found the EAP by searching independently, others accessed the service on, or assisted by**,** the personal recommendations of someone trustworthy (see Table 3).

I just remember going on the intranet and searching for, like, ‘[company name] counselling’ or something, and then the EAP came up, I went from there and then actually spoke to one of the partners in my group and he recommended it. (5)

As an extension of this personal recommendation, one participant who had himself been recommended to use an EAP, now offered himself as evidence that its use had no stigma attached. This was, for him, a key message in getting other men to use it.

The hardest part… is kind of helping our juniors get the message that there is no stigma attached to it… I’m quite quick when I have a discussion with them to say, “I’ve used the service and senior people knew that I did”. I’m [in a senior position]. I feel like I’m having reasonable success and it hasn’t affected me.” So, I think it’s quite a key message. (3)

### Motivators and enablers of potential EAP use

This section reports on what the participants say would potentially enable or motivate them to use an EAP and could be framed as building trust. Not surprisingly, many suggestions are potential solutions to the barriers they identified, and which are reported earlier in the results. Solutions address issues of uncertainty as to what sort of service is being delivered, by whom, and the career consequences of EAP use. The motivators and enablers were categorised as: (a) more attractive, relatable, and trustworthy messaging, and (b) proactive service delivery (see Fig. 3).

#### More attractive, relatable, and trustworthy messaging

This messaging included: (a) more factual detail as to the specifics of the service, (b) messaging that was less “warm and fuzzy’’ (blue-collar), (c) in-house male leaders as Brand Ambassadors and Champions (white-collar) and lastly, (d) market EAP as providing a pro-active competitive advantage (white-collar).

*More factual detail* Both white-collar and blue-collar participants wanted messaging to provide more factual detail about the service***.*** This could include its professionalism, modes and hours of access and cultural sensitivity.

Something we need to really push [is] that these people [EAP counsellors] are trained.… These people… are professionals. You don’t get your mate to fix your teeth. So, don’t get your mate to … try and fix your head. (7)

Participants also wanted messaging to show examples of issues that warranted help-seeking from EAP services. Additionally, white-collar workers advised that they had to deal with an overwhelming amount of information daily, so EAP information needed to stand out and be differentiated from other types of wellness messages.

Advertise specific examples of the problems [that] people do come to [EAP provider] with just to show that no issues are too small or too big to take to them…. make it more relatable… there are people with the same issues as me that actually go and use these services; maybe I’ll do it too. (12)

*“Less Warm and Fuzzy”* Blue-collar workers said they would be more attracted to the service if they perceived it as, “less warm and fuzzy.” This subtheme relates to the workers’ perception of counselling as soothing passive talking that does not actively progress a solution.

You look at men's sheds or …motorbike clubs - men get together they do talk about things, but because it's a butch environment it's not warm fuzzies what men do together. And they do eventually talk, but they don't necessarily go to the nice warm fuzzy solution because it's not what we do. (10)

*In-house male leaders as brand ambassadors* The white-collar workers said they would trust a service more if they knew it could be used without stigma or career jeopardy. They suggested having in-house male leaders as brand ambassadors and champions for men’s mental health. Brand Ambassadors could be senior male leaders from the organization who had accessed an EAP and were willing to publicly talk about that experience; men that other men could relate to. This would be a particularly powerful endorsement of the service.

It also just needs a champion, somebody to step up and say my masculinity is not threatened by this. Part of my masculinity is owning where I’m at. We need someone to stand up and be that. (7)

*Proactive competitive advantage* The white-collar corporate environment is competitive, “dog-eat-dog’’ (9) as one participant described it. Men had to stand out from their peers if they wanted to succeed. Participants suggested marketing the EAP as proactive agents, as providing employees with additional skills and thus providing a competitive advantage.

You know if we introduce [EAP] initially “Hey, here’s how you can build some resilience, which is another skill that you have, which is a good thing,” people are taking it in, the macho element will all fit into it, people will have an extra ability or an extra skill or an extra something or other. (1)

#### Proactive approach to service delivery

In addition to making messaging more attractive and relatable, participants also suggested that EAP providers take a proactive approach to service delivery. They suggested: (a) creating a connection before an issue arose (white- & blue-collar), (b) providing a mental health toolbox, and (c) mandating employee contact with EAP (white-collar).

*Connection before an issue arises* Both white- and blue-collar participants said they would be more comfortable accessing a service when a problem arose if they had established a prior connection. This would build a sense of trust. Many blue-collar participants said they felt uncomfortable talking about their problems with people they did not know. If an EAP created a bond with the workers, this would help build trust.

I'm sorry, but they won't [open up] until they [EAP representatives] create a bond with them… but it's getting them to go from not knowing an outfit like yours to knowing them, that’s going to be the hardest part to happen. But once that occurs it's no problem; they’ll talk to you about anything and everything…It's just a manly thing, they don't automatically trust anybody. (11)

In a similar vein, the white-collar workers suggested that men could try out the service, by calling the number and talking to a counsellor before they felt any real need to use it.

Encourage men to try out the service with something that’s not so dramatic just to see what comes out of it because maybe that flushes-out another bigger topic. (2)

*Mental health toolbox* Some white-collar participants also suggested integrating the EAP more closely with other specific mental health strategies within the workplace, so it provided resources, not just for problem resolution, but for improving mental health awareness and education. They suggested an EAP provide workers with self-help skills, like tools in a toolbox—effectively a mental health toolbox**.** This provision of skills could also tap into the male preference to ‘do-it-yourself'. Self-help skills could be promoted as useful not only for the individual, but also as a tool to help others.

So, if there’s tools that can be taught [so] that you can manage these things on your own…. that sort of stuff resonates a lot more with boys because that's what we love, right? We love being able to solution and tool it ourselves. (9)

*Mandate EAP sessions* A few white-collar participants also suggested that, in addition to the EAP provider delivering more general skills training, their employer should mandate sessions with the EAP provider on a one-to-one basis. This would demonstrate the organisation’s commitment to men’s mental health in that men would be given the time and space to discuss personal issues.

An hour a week where the first half hour is: these are the skills that we're educating today… Then the next half is “feel free to stay if you'd like to talk about things that are causing you angst at the moment.” Yes, and if you don't have an issue, you have to speak, you have to sit down, “tell me about what's going on.” (9)

## Discussion

This exploratory study sought contemporary evidence that would help us better understand why men do not seek help from their EAP service. Many aspects of the barriers reported by our participants had been previously reported in studies that were conducted in several countries and that date back to 1988 (e.g., [29, 51]): general lack of knowledge about the service, stigma associated with its use, issues of trustworthiness and confidentiality, and potential for negative impact on career paths. This important finding indicates that some barriers to EAP use are more entrenched than were previously estimated, and that previous efforts to overcome them have been, in retrospect, somehow ineffective, misdirected or perhaps as yet unaddressed. We could find only one recent study that specifically addressed barriers to EAP use and it focused on leader mental health training [52]. The lack of specific research evaluating improvements to EAP delivery and subsequent uptake by employees suggests that improving uptake is not a priority for client organisations over and above being able to recommend a cost-effective support resource for staff, having a risk mitigation strategy, and meeting an industry expectation [53].

Notably, a lack of knowledge about EAPs and knowing when to use EAP services are enduring barriers to its use (see [30, 33, 51, 54]). On face value, it may be easy to consider these enduring barriers as solely arising from poorly targeted EAP marketing, however contributing causes may also be found at the EAP provider and client organisation level. Human Resource professionals frequently lack an understanding of EAPs and their services [34, 53], while others point to the less than optimal ‘robust and effective’ communication that is required between EAP providers and client organisations to deliver optimal EAP promotion and development [18]. The literature did not reveal any study that had examined the nature of ‘EAP provider–client organisation-employee’ interactions and their impact on EAP use. This research is needed to identify optimal pathways to the delivery of strong, clear, and consistent messaging about EAPs and their services.

Strong, consistent messaging also needs to be relatable. Rethinking the language that is used in traditional ways of help-seeking may also benefit EAP brand promotion. The men in our study preferred active problem solving to solutions utilising talk therapies, which they perceived were offered by EAPs. Reshaping marketing to present EAPs as skill development, akin to the skill toolboxes described in the focus groups, and/or providing coaching and resilience training for a competitive edge, are likely to be more attractive messages. Examples provided by practitioners in Mahalik et al.’s study [55] are particularly salient and reflect the examples from our study: “Call it consultations or coaching for traditionally socialized men” or “consider alternative forms of therapy (psychoeducation, workshops, videos) to reach men that are not comfortable with traditional therapy” or to “frame therapy as an educational opportunity” (p. 597). This use of language would help to engage men who want skills to help themselves but that can also be used to gain competitive advantage.

Our study also aimed to identify differences in the views of men working in blue- and white- collar organisations in order to identify meaningful ways to tailor EAP marketing and support services to these cohorts of workers. To our knowledge our findings provide initial evidence on this topic. Organising our findings into a schema provided a visual appreciation of the barriers and enablers to EAP use, as well as differences between the two types of workers’ groups.

A prominent obstacle specific to EAP use for white-collar participants was the overall distrust of the corporate motives in providing EAP mental health and wellbeing services. EAPs are suggested as being a key business strategy that organisations purchase to improve job performance [56]. Increasing employees’ workloads while at the same time providing EAP services for use by employees who subsequently experience difficulties with work and productivity, was viewed as unjust. Purchasing EAP support services to ensure employee problems had minimal disruption to productivity also contributed to the lack of trust the men had in the autonomy and confidentiality of the service. Having a free support service provided in a work context of increasing workloads raised questions about the independence and quality of the advice that would be received. Increased understanding is needed by EAPs and their white-collar client organisations of this perceived paradox.

Further work is needed to identify the best way for the promotion of employer purchased EAP services to sit comfortably alongside its users’ employment expectations, work culture and work values. Organisational theory provides some approaches to guide this work. For example, Rousseau’s [57] work on psychological contracts between employees and their managers provides a framework with which to examine employees’ perceptions of fairness when EAP is offered in response to increased workload and related psychosocial hazards—and the impact their perceptions have on the trust that underpins the contracts. At an organisational level, the work by Lewicki and colleagues [58–60] on trust in organizations provides ways for organizations to develop, sustain and repair trust in professional work relationships.

Our white-collar participants strongly endorsed using EAP brand ambassadors—a culture and leadership model—as one way to begin to address distrust. Ambassadors would be successful men from their workplace who had accessed the service without career detriment and who could take an ongoing role that promotes the benefits of EAP services. Openly talking about mental health has been used frequently in mass media advertising and community education to reduce stigma [61, 62] and when done by ‘one of their own’ may provide corporate men the needed reassurance they seek in order reach out to EAP services. For EAP to be taken seriously as a resource to assist men’s wellbeing, our white-collar participants also suggested that there would be value in men attending regular mandatory EAP sessions. Doing so would be a valuable way to remove the stigma associated with accessing the service for those men who needed help with psychosocial issues, while also providing all men with an opportunity to gain problem solving, coaching and other career advancing skills.

Both groups of participants viewed what the men described as a ‘deal with it yourself’ culture, where emotional toughness and resolving problems individually was a strong male virtue, as a major barrier to EAP use. An additional barrier for white-collar participants was the extension of this virtue to the corporate environment, the strong male virtue that demonstrated capability in a peer or leader. As discussed above, men’s use of mental health support is still strongly associated with stigma [36, 63, 64], and our participants, as well as employees in previous studies [33, 65], had concerns of being stigmatized by management if their use of EAP services was revealed. It would appear that despite legislation that makes discrimination unlawful (e.g. Australian Disability Discrimination Act 1992 [66], Fair Work Act 2009 [67]), anti-stigma training for employees and their co-workers [68] and workplace based training to increase managers’ understanding of mental health concerns [69], many behaviours go unregulated [9] and men are still hesitant to seek help from EAP services.

In the absence of a positive change to workplace culture that removes the stigma associated with help-seeking, EAP initiatives that explore ways to provide services to men through more direct means may help increase their access to services. Although the use of information technology was not raised as a mechanism to reduce the barriers to EAP by the men in this study, the potential for its use in the delivery of EAP messaging and services offers some food for thought. Smartphone apps, in particular, have improved men’s accessibility to mental health information and services in other settings [70]. Additionally, the use of chat bots has gained attention as a good way to provide psychoeducation, to offer anonymity, and reach people reluctant to reach out for mental health advice because of concerns they have of being stigmatized [71].

In addition to workplace stigma that participants reported as thwarting their help-seeking, many of our white-collar participants believed that they were characterised by their organisation as being privileged and therefore not needing individualised mental health support—a perception consistent with the World Health Organisation’s views on gender-based disparity in health [72]. Men in general enjoy more opportunities, privileges, and power than women; however, in the context of help-seeking, this privilege does not translate into better health. Rather, the persisting assumption that men are disinterested in their health has actively discouraged men’s use of health services [73]. Our findings suggest there is real potential for EAP providers to work with client organisations to improve the “men’s health gap” [72] and to design and provide easier pathways to mental health support for men.

## Strengths and limitations

Male employees self-selected to participate in focus groups, and even though these groups included representatives of all levels of management, these men may have differed to other men in the participating organisations. The findings from this study may have limited generalisability to other workplace environments as only two organizations were utilized in this study. Qualitative research, however, does not intend to be representative, rather, it aims to provide a deep understanding of context issues, perceptions and meanings [74]. Having fluent English as a requirement to participate may have disadvantaged some men’s ability to contribute, although probably less so for the white-collar workers where fluent English is required to conduct business. Having females moderating men’s focus groups and asking about a sensitive issue such as men’s use of mental health services, may have influenced group behaviour or the articulation of certain views by some participants. On the other hand, moderators were experienced with facilitating focus groups and their dynamics, and one moderator was known to some participants which enabled rapid rapport and openness in the men’s discussions. The need for confidentiality was stated at the outset of each group or interview as an expectation. But some men may have been reticent to speak freely because of concerns they had that other participants may not maintain such confidentiality once the groups adjourned. Additionally, a group bias effect may have influenced by the dynamics of the group, and group consensus, making it less likely for participants with divergent opinions, to express those opinions within the group setting. These aspects considered, we consider our findings and their interpretation to be trustworthy. Each author contributed their expertise to the analysis and interpretation, and this comprised extensive experience in qualitative methods, three different professional frameworks, and professional experience as mental health care workers.

## Conclusion

Published literature that reports men’s perspectives of barriers to and enablers of the uptake of workplace based EAP services in Australia is scant. Our exploratory study provided a contemporary account of men’s perspectives on these help-seeking issues. The finding of enduring barriers to EAP use is concerning given the wide-spread use of EAP services by organisations in Australia and other countries. It suggests the need for a rebranding of EAPs to establish credibility with the end users of the service—employees. Our findings on the differences in views held by men from blue- and white-collar workplaces are novel and point to the ways that EAP providers can advance their programs and tailor their marketing to better attract men from blue- and white-collar work environments. Tailored marketing is an important consideration in light of the high rates of suicide experienced by men [2] and the differential gradient of suicide between high-skill and low-skill occupations [40].

Research that examines the impact of rebranded and tailored EAP marketing to men, the impact of intentional changes to work culture to improve trust, men’s actual uptake of EAP services is now needed. An ongoing engagement with men about the suitability of EAP services to their needs is central to maintaining the value of the services EAP offers men. Increasing men’s uptake of EAP services can not only lessen the emotional burden on the individual and help reduce the incidence of suicides in men, but also reduce the flow-on psychosocial and financial impact to family, friends, workplace and the wider community. This is particularly so in the context of COVID-19 which has abruptly disrupted many workplaces and work practices and has adversely affected mental health [75]. This study was conducted before COVID-19 and although the need for workplace mental health services will increase due to COVID-19 [76], its impact on EAPs and their modes of service delivery will need further research. While acute impact research is being published (e.g. [77]), the longer term impacts are as yet unknown.

## Data Availability

Data cannot be shared publicly as participants did not give consent for their transcripts to be shared in the public domain. Data are available from the University of Sydney’s Human Research Ethics Committee (contact via human.ethics@sydney.edu.au) for researchers who meet the criteria for access to confidential data.

## Declarations

### Authors’ Information

JG is now located in London, United Kingdom and her contact email is Jackygerald@aol.com.
